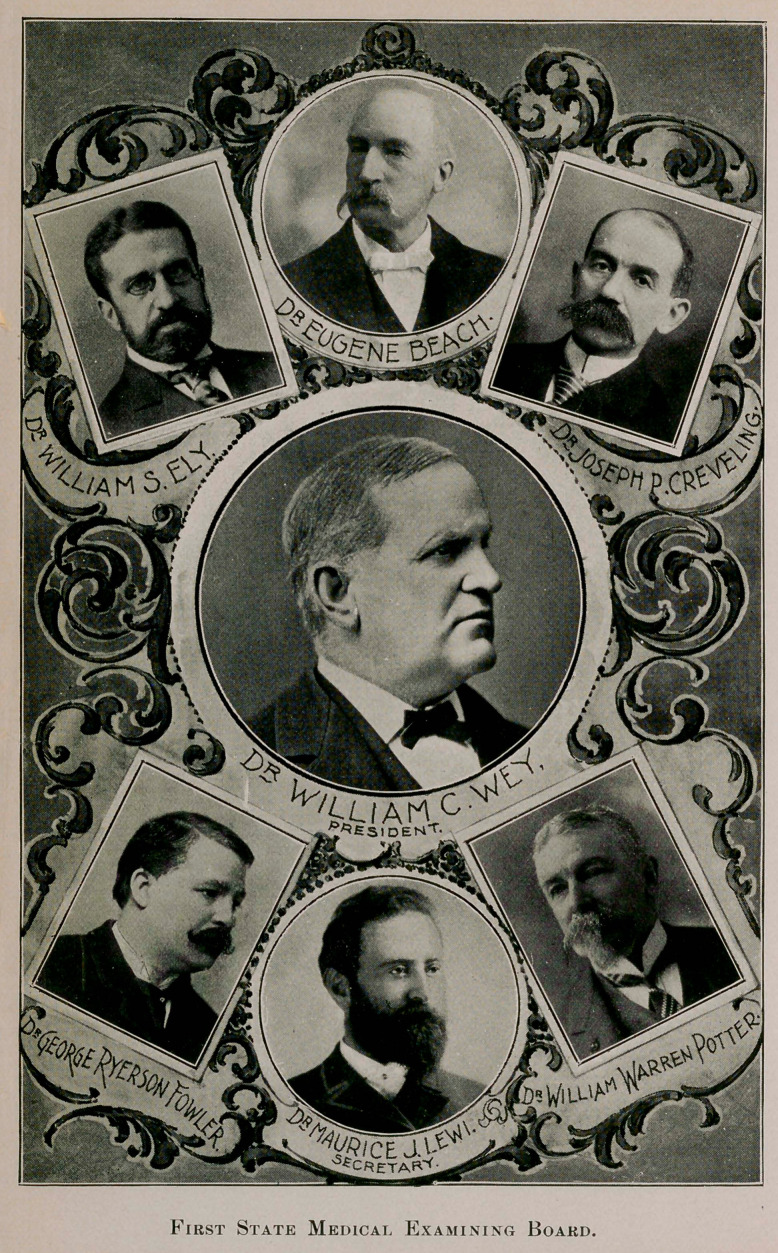# Street Noises

**Published:** 1898-01

**Authors:** 


### Selection.

### STREET NOISES.

DOES the average practitioner ever think of or take into account the various noises that obtain in a large and thickly inhabited community to the general detriment of the ill and nervous ?

Many among healthy individuals, of course, become so accustomed to street noises that they excite no comment and hence do not in the least constitute a disturbance ; and yet these same individuals stretched upon a sick bed may become very sensitive thereto. In the stone-paved streets of the cities of Great Britain it is not uncommon to see straw distributed with lavish hand for a block or more, with no other purpose in view than deadening the sound of passing wheeled vehicles that might otherwise disturb the rest of some invalid ; but in the United States such an act would probably bring down the anathemas of the neighborhood, perhaps even to "getting one into trouble with the police."

Where electric cars are continually passing, with the constant rattle of the gong as manipulated by the motorman, the jangling of the bell that conveys the signals of the conductor, coupled with the pounding of the wheels upon the rail and hissing of the trolley upon the wire, these, singly and collectively, constitute one of the most exasperating of the worries of the afflicted, particularly in summer, when the windows are perforce left open to secure the desired amount of fresh air and coolness. Think of the invalid subjected to this once every five to seven minutes !

Then there are the street cries ; and thrice daily the screeching of the whistles and jangling of great bells ; the crash of rapidly moving fire engines ; the howling blasts of railroad engines and of boats—all tending to make a pandemonium that oftentimes is all but unbearable to one already half-demented as the result of high temperature and burning fever.

Is it not fully time that some of these barbarisms were done away with ? The street cars, of course, cannot be governed any more than can the rattling obtaining to the movements of fire engines and vehicles—at least not until there is a radical change in the arrangement of wheels, and perhaps in rails and pavements as well. Some cities have already set a good example in attempts to suppress these nuisances. Indianapolis, for instance, has forbidden the blowing of whistles and horns within the city limits.

Proper municipal reform will embrace not only the foregoing, but lead to suppression of all ringing of bells in church steeples and upon engines, all blowing of whistles as regards factories, steamers and locomotives. The church bell is a medieval relic that does not in the least tend to inculcate Divine precepts—rather the contrary! It might be tolerated if there was any good reason therefor, or even if there was any unison or suggestion of harmony; but instead noise and evidence of money expended alone seems to be the ends in view. The church-goer certainly does not depend upon iron tongues and brazen throats to inform him of the hours of service. Neither does the factory-hand require a whistle to call him or her to their duties—the check window and timekeeper are far more effective. Are there, then, any cogent reasons why both these nuisances should not be forever suppressed, not only in the interests of the ill, but as a means of ensuring rest and comfort to the community at large?

Again, the railway whistle might easily be replaced by a bit of the block system of rail, which would convey the desired information to the watchman at the crossing, or to the agent, by electricity. So, too, the locomotive bell in the same way might be replaced by electric gongs fixed at each and every crossing.

Surely there is no excuse in these days of flag-signals, telephones and telegraphy, for the prolonged sounding of whistles by steamers, the sole purpose of which is to inform owners that their property is passing—a bit of information that could better be conveyed by the marine reporter through telephone or over wire, and that, moreover, is almost invariably chronicled in the morning and evening papers; neither is the prolonged three blasts that heralds the approach to a wharf at all essential. Let these things be positively forbidden except in cases of fog or to signal approaching craft to designate the course to be steered. As already remarked, the majority of these nuisances arise from the desire to make a noise; and it is solely for this reason that every newly-built house of worship in a small community attempts to secure a larger and louder bell than its neighbors. These noises in the large cities on some days, or on parts of days, are almost continuous.

Truly this is a matter it behooves medical men to look after and bring before the municipal authorities of every large community with a view of permanently suppressing, or at least in a great measure mitigating. The regulation of whistling by steamers on large waterways, however, may perhaps require the consent and legislation of the national government, but even that, considering the object to be gained, should be far from a Herculean task.—*Medical Age.*

## Figures and Tables

**Figure f1:**